# Change in dietary intake of adults with intermittent claudication undergoing a supervised exercise program and compared to matched controls

**DOI:** 10.1186/1475-2891-13-100

**Published:** 2014-10-15

**Authors:** Christopher L Delaney, Michelle D Miller, Kacie M Dickinson, J Ian Spark

**Affiliations:** Department of Vascular Surgery, Flinders University, Adelaide, Australia; Department of Nutrition and Dietetics, Flinders University, Adelaide, Australia

**Keywords:** Peripheral arterial disease, Diet, Nutrition, Supervised exercise, Claudication

## Abstract

**Background:**

Presence of numerous diet responsive comorbidities and high atherosclerotic burden among adults with intermittent claudication demands attention is given to diet in an effort to delay progression of peripheral artery disease. The aim of this study was to compare diet of adults with intermittent claudication: (a) against dietary recommendations; (b) following 12 weeks of supervised exercise training; and (c) against non-peripheral artery disease controls.

**Methods:**

Diet was assessed using a food frequency questionnaire pre and post supervised exercise training. Pre-exercise diet was compared against Suggested Dietary Targets and against non-peripheral artery disease controls matched for gender, age and body weight. Pre-exercise diet was also compared against post-exercise diet.

**Results:**

Pre-exercise 25/31 participants, 5/31 participants, 16/31 participants and 4/31 participants achieved recommendations for protein, carbohydrate, total fat and saturated fat respectively. Few achieved recommended intakes for fibre (3/31 participants), cholesterol (8/31 participants), folate (11/31 participants), potassium (1/31 participants), sodium (4/31 participants), retinol equivalents (1/31 participants) and vitamin C (3/31 participants). There were no differences observed between participants compared to controls in achievement of recommendations. Post-exercise, marginally more participants were able to achieve targets for cholesterol, sodium and vitamin C but not for any other nutrients.

**Conclusions:**

Despite evidence to support benefits of dietary modification in risk reduction of peripheral artery disease, adults with intermittent claudication continue to consume poor diets. Research is required to determine whether dietary changes can be achieved with greater attention to nutrition counselling and the impact assessed in terms of delayed disease progression and long term health outcomes.

**Trial Registration:**

ClinicalTrials.gov: NCT01871779.

## Background

Peripheral arterial disease (PAD) is a local manifestation of the diverse pathophysiological processes associated with the systemic disease state atherosclerosis. The pro-inflammatory and pro-oxidative nature of the disease together with resultant immobility and impaired quality of life precipitates a nutritional vulnerability in these patients which may compound an already increased risk of coronary and cerebrovascular disease and subsequently premature death [1]. The nutritional vulnerability of these patients takes extra significance when one considers that current international consensus guidelines recommend a supervised exercise program as a treatment for all patients with PAD manifesting as symptoms of intermittent claudication but gives little attention to nutrition education and counselling [2].

Peripheral arterial disease ordinarily co-exists with numerous diet-responsive cardiovascular and metabolic comorbidities such as hypertension, hyperlipidaemia and diabetes mellitus [3]. It is well documented that dietary modification can delay progression of atherosclerosis [4] and that some nutrients appear to be protective against the development of PAD [5]. For those with PAD, there is increasing epidemiological evidence that single nutrients such as carnitine [6] or multi-nutrient combinations [7] may facilitate improved outcomes. Despite this array of evidence, the dietary intake of adults with PAD has not been extensively investigated.

In the only relevant study Gardner and colleagues [8] compared the dietary intake of 46 males with PAD of varying severity with accepted dietary recommendations and observed that none were able to achieve the recommendations for sodium and vitamin E, and only a small percentage were able to achieve the recommendations for folate (13%), saturated fat (20%), fibre (26%) and cholesterol (39%). The authors concluded that adults with PAD have poor diets. This is despite there likely being several teachable moments during progression of the disease for dietary advice to be provided.

Extensive evidence demonstrates regular exercise is favourable for cardiovascular health and can improve walking performance in patients with IC [9, 10]. Unlike diet, exercise is routinely recommended for conservative management of adults with PAD manifesting as IC, appearing as a key recommendation in commonly adopted clinical practice guidelines and systematic reviews [2, 11]. From a nutritional perspective, the impact of such an intervention has not yet been established but may be balanced by two contrasting outcomes. Firstly, the increase in metabolic demands associated with exercise [12] may lead to a decline in nutritional status and physical health and further highlights the importance of nutritional status in this vulnerable group of patients. Secondly, given that in other settings, exercise training may improve dietary composition even in the absence of nutrition counselling [13–15] it could be speculated that a spontaneous improvement in dietary intake might occur and provide an additive benefit to exercise therapy for adults with IC. If exercise can result in optimising dietary intakes then benefits might include delayed progression of PAD and improved management of co-existing chronic disease risk factors.

The aim of this study was to compare usual dietary intake of adults with IC: (a) to suggested dietary targets; (b) following 12 weeks of SET; and (c) against non-PAD controls.

## Materials and methods

### Subjects

22 men and 9 women aged ≥49 years with IC were recruited from the Southern Adelaide Local Health Network Vascular Surgery Claudication Clinic to participate in a 12 week supervised exercise training (SET) intervention designed to maximise walking performance. The SET ran over 12 weeks and consisted of two 60-minute sessions per week supervised by a senior physiotherapist. The SET included treadmill-based training with or without resistance training and is described in detail elsewhere [16]. In addition the participants received counselling on cardiovascular risk factor and lifestyle modification by the medical staff (smoking cessation, regular exercise, healthy diet, weight loss) and attended an educational lecture series, including a 30 minute seminar on healthy diet for PAD. Patients attending the Claudication Clinic with a clinical history consistent with IC, ankle brachial pressure index (ABPI) <0.9 in any ankle artery and radiographic evidence of infra-inguinal disease in the absence of aorto-iliac disease were eligible for inclusion. Those attending the Claudication Clinic who had experienced lower limb ischaemic rest pain, had clinical evidence of tissue loss such as ulcers or necrotic lesions, had undergone arterial intervention (endovascular or open surgery) in the preceding 12 months, suffered from pre-existing cardio-respiratory morbidities limiting exercise capacity, had evidence of aorto-iliac disease or were deemed to be not competent of providing written informed consent were not considered eligible for participation in the study. Ethics was approved by the Southern Adelaide Clinical Research Ethics Committee.

### Demographics, medical history and clinical characteristics

Comorbidities, current medications, smoking status and demographic data were recorded from medical records. Walking performance was measured using a six-minute walk test, demonstrated as highly reliable and related to the functional and hemodynamic severity in patients with IC [17]. Pain free walking distance was recorded as the distance, in metres, at which participants first reported claudication pain. Body weight and height were measured to 0.05 kg and 0.1 cm using digital scales and a wall-mounted stadiometer respectively, while wearing light clothing and no shoes.

### Dietary intake

Energy and nutrient intake for all participants was assessed using the validated 74-item Dietary Questionnaire for Epidemiology Studies Version 2 (DQES v2) [18] pre and post-SET. Validation of the DQES v2 has demonstrated that it can capture similar nutrient intake data as the more burdensome weighed food records and may be used for estimation of dietary intakes over a relatively short time in clinical intervention trials [19].

The DQES v2 contains 74 food items with 10 frequency response options ranging from ‘never’ to ‘3 or more times per day’ in addition to photographs of scaled portions for four foods used for calibration of portion size. There are also questions for calibration purposes on the overall frequency of consumption of selected fruits and vegetables and consumption of other selected foods that do not fit easily into the frequency format. The food composition data used to calculate nutrient intake are derived from *NUTTAB95* [20], with supplementation of other data where necessary [21–24].

The Australian and New Zealand Acceptable Macronutrient Distribution Range and Suggested Dietary Targets (SDT’s) for lowering chronic disease risk [25] were selected for the dietary intake of participants to be compared against. For energy, individual estimates of total energy expenditure (TEE) were determined according to the equation of Schofield [26] with adjustment for a physical activity level of 1.4 which is considered as very sedentary according to NHMRC [25]. The ratio of energy intake to TEE <0.79 was applied as a cut-off value to define energy under-reporters. This value is consistent with the lower 95% confidence limit of the ratio of energy intake to TEE, where TEE is measured directly by doubly labelled water [27]. For alcohol, <2 standard drinks (<20 g alcohol) daily reduces the lifetime risk of harm from alcohol-related disease or injury according to the NHMRC and hence this was the recommendation applied for the purpose of this study [28].

The dietary intake of participants was also compared against a gender, age (±5 years) and body weight (±5 kg) matched sample of adults with no history of PAD. The non-PAD control sample were participants of a vascular health screening program who were assessed as not having PAD according to ABPI >0.9 and completed the same DQES v2 as those with IC.

### Statistical analyses

All values are reported as mean ± SD and n (%) for continuous and categorical data respectively. To compare energy and nutrient intake of those with IC pre and post SET, and separately nutrient intake of those with IC pre-SET against a gender, age and weight matched sample of adults without PAD, paired samples t-tests were applied. Similar comparisons were made according to the proportion achieving the AMDR, SDTs and alcohol intake recommendations using the chi-square or Fishers exact test of association. Significance was set at *P* < 0.05 and all analyses were conducted using IBM SPSS Statistics Version 20.

## Results

### Subjects

Participant demographics and clinical characteristics pre-SET are summarised in Table 1. The majority of the sample were male (n = 22, 71%), almost half were smokers (n = 14, 45%) and most experienced established risk factors for PAD including hyperlipidemia (n = 26, 84%) and hypertension (n = 26, 84%). The non-PAD controls (n = 23) had similar BMI (27.2 ± 4.3 kg/m^2^) to the participants with PAD, more were smokers (n = 14, 61%), but fewer (n = 9, 39%) had established hyperlipidemia or hypertension (n = 9, 39%).

**Table 1:** **Clinical characteristics of participants with peripheral arterial disease and intermittent claudication pre-commencement of 12 weeks supervised exercise training, n = 31**

Variables	
Mean (SD) Age, years	70.7 (10.0)
Gender, n (%) male	22 (71)
Mean (SD) Pain Free Walking Distance, metres	157.9 (93.5)
Current smoking, n (%) yes	4 (12.9)
Diabetes, n (%) yes	10 (32.3)
Hypertension, n (%) yes	26 (83.9)
Dyslipidemia, n (%) yes	26 (83.9)
Mean (SD) Body mass index, kg/m^2^	28.0 (4.8)

### Dietary intake of participants with IC

The mean (SD) macronutrient and micronutrient intake of participants with IC pre-SET is presented in Table 2. Mean (SD) dietary energy intake was 7837 (3111) kJ/day and 13 (42%) were identifiable as under-reporters of energy intake. While the majority of participants achieved the AMDR for protein (n = 25/31, 81%), there were few participants achieving the AMDR for carbohydrate (n = 5/31, 16%), total fat (n = 16/31, 52%), saturated fat (n = 4/31, 13%), polyunsaturated fat (n = 3/31, 10%) and none achieved the AMDR for monounsaturated fat. Similarly, there were few participants who achieved the SDTs for fibre (n = 3/31, 10%), cholesterol (n = 8/31, 26%), folate (n = 11/31, 36%), potassium (n = 1/31, 3%), sodium (n = 4/31, 13%), retinol equivalents (n = 1/31, 3%), vitamin C (n = 3/31, 10%) and none that achieved the SDT for vitamin E.

**Table 2:** **Dietary intakes of participants with peripheral arterial disease and intermittent claudication prior to commencing 12 weeks supervised exercise training (SET) and n (%) meeting Australian recommendations** ^**a**^ **in comparison with gender, age (±5 years) and weight (±5 kg) matched non-PAD controls**

	Mean (SD) IC pre-SET (n = 31)	AMDR or SDT ^a^	n (%) achieving AMDR or SDT ^a^		
			IC pre-SET (n = 23)	Non-PAD controls (n = 23)	P value
Protein, g/day	90 (39)				
*% kJ from protein*	20 (4)	15-25% TE	18 (78)	18 (78)	1.00
Carbohydrate, g/day	194 (81)				
*% kJ from carbohydrate*	40 (6)	45- 65% TE	4 (17)	4 (17)	1.00
Total fat, g/day	74 (32)				
*% kJ from total fat*	37 (5)	20-35% TE	15 (65)	9 (39)	0.179
Saturated fat, g/day	29 (13)				
*% kJ from SFA*	13 (3)	<10% TE	4 (17)	0 (0)	na
Monounsaturated fat, g/day	27 (12)				
*% kJ from MUFA*	12 (2)	>20% TE	0 (0)	0 (0)	na
Polyunsaturated fat, g/day	13 (7)				
*% kJ from PUFA*	6 (2)	8-10% TE	2 (9)	1 (4)	1.00
Cholesterol, mg/day	300 (131)	200	2 (9)	2 (9)	1.00
Sodium, mg/day	2592 (1257)	1600	3 (13)	1 (4)	1.00
Potassium, mg/day	2923 (997)	4700	0 (0)	1 (4)	na
Folate, μg/day	284 (109)	300	8 (36)	7 (32)	0.343
Fibre, g/day	M: 24 (8) F: 21 (8)	M: 38 F:28	2 (9)	2 (9)	1.00
Retinol equivalents, μg/day	M: 918 (311) F: 771 (242)	M: 1500 F: 1220	0 (0)	1 (4)	na
Vitamin E, mg	M: 7 (3) F: 5 (2)	M: 19 F: 14	0 (0)	0 (0)	na
Vitamin C, mg	M: 119 (70) F:120 (39)	M: 220 F: 190	3 (13)	3 (13)	1.00

*Abbreviations*: *AMDR* Acceptable Macronutrient Distribution Range, *F* female, *M* male, *MUFA* monounsaturated fat, *na* not applicable, *PUFA* polyunsaturated fat, *SDT* Suggested Dietary Target, *SET* Supervised Exercise Training, *SFA* saturated fat.

^a^Recommendations according to the National Health and Medical Research Council Nutrient Reference Values for Australia and New Zealand [25].

Table 2 highlights that when participants with IC (n = 23/31) achieving the AMDRs were compared to non-PAD controls, there was no statistically significant difference observed for protein, carbohydrate, total fat, saturated fat, polyunsaturated fat or monounsaturated fat. Similarly, there was no statistically significant difference in the number of participants with IC able to achieve the SDTs for cholesterol, fibre, folate, potassium, sodium, retinol equivalents, vitamin E or vitamin C compared to non-PAD controls. For energy intake, mean (SD) was 7642 kJ (2256) pre-SET compared to non-PAD controls 8260 kJ (2580), P = 0.287 and there was no significant difference in the prevalence of underreporting energy intake between participants with IC (n = 10/23, 44%) and non-PAD controls (n = 6/23, 26%), P = 0.341.

Table 3 highlights that the number of participants with IC achieving the AMDRs pre-SET compared to post-SET were not significantly different for protein, carbohydrate, total fat, saturated fat, polyunsaturated fat or monounsaturated fat. For energy intake, mean (SD) was 7676 kJ (3143) pre-SET compared to 7931 kJ (3974) post-SET, P = 0.693. There were no statistically significant differences in the number of participants with IC able to achieve the SDTs pre-SET compared to post-SET for fibre, folate, potassium, retinol equivalents, or vitamin E. Post-SET, there was an increase in the number of participants with IC able to achieve the SDTs for cholesterol (n = 9/29, 31% vs n = 8/29, 28%), sodium (n = 7/29, 24% vs n = 4/29, 14%) and vitamin C (n = 4/29, 14% vs n = 3/29, 10%).

**Table 3:** **Dietary intake of participants with peripheral arterial disease and intermittent claudication pre and post-commencement of 12 weeks supervised exercise training (SET)**

	Pre-SET ^a^		Post-SET ^b^		P-value ^b^	P-value ^c^
	Mean (SD)	n (%) achieving AMDR or SDT ^a^	Mean (SD)	n (%) achieving AMDR or SDT ^a^		
Protein, g/day	88 (40)		94 (52)		0.510	
*% kJ from protein*	20 (4)	23 (79)	20 (4)	27 (93)		0.377
Carbohydrate, g/day	189 (81)		189 (82)		0.962	
*% kJ from carbohydrate*	39 (6)	5 (17)	40 (6)	5 (17)		1.00
Total fat, g/day	73 (32)		77 (43)		0.550	
*% kJ from total fat*	34 (5)	16 (55)	34 (3)	16 (55)		1.00
Saturated fat g/day	28 (13)		29 (17)		0.619	
*% kJ from SFA*	13 (3)	4 (14)	13 (3)	4 (14)		1.00
Monounsaturated fat g/day	26 (12)		28 (17)		0.491	
*% kJ from MUFA*	12 (2)	0 (0)	12 (2)	0 (0)		1.00
Polyunsaturated fat g/day	12 (7)		13 (8)		0.606	
*% kJ from PUFA*	6 (2)	3 (10)	6 (2)	4 (14)		0.371
Cholesterol, mg/day	299 (133)	8 (28)	309 (166)	9 (31)	0.740	0.004
Sodium, mg/day	2537 (1263)	4 (14)	2568 (1474)	7 (24)	0.910	0.001
Potassium, mg/day	2818 (944)	1 (3)	2866 (1186)	1 (3)	0.806	1.00
Folate, μg/day	279 (110)	9 (31)	267 (102)	9 (31)	0.549	0.088
Fibre, g/day	M: 24 (8) F: 20 (9)	3 (10)	M: 25 (8) F: 18 (9)	1 (3)	M: 0.590 F: 0.206	0.103
Retinol equivalents, μg/day	M: 896 (301) F: 756 (254)	1 (3)	M: 815 (298) F: 662 (376)	1 (3)	M: 0.275 F: 0.218	1.00
Vitamin E, mg	M: 7 (3) F: 5 (2)	0 (0)	M: 7 (3) F: 5 (3)	0 (0)	M: 0.151 F: 0.325	na
Vitamin C, mg	M: 117 (71) F: 119 (42)	3 (10)	M: 132 (65) F: 107 (45)	4 (14)	M: 0.945 F: 0.521	0.001

*Abbreviations*: *AMDR* Acceptable Macronutrient Distribution Range, *F* females, *M* males, *MUFA* monounsaturated fat, *na* not applicable, *PUFA* polyunsaturated fat, *SDT* Suggested Dietary Target, *SET* Supervised Exercise Training, *SFA* saturated fat.

^a^Recommendations according to the National Health and Medical Research Council Nutrient Reference Values for Australia and New Zealand – see cut-offs in Table 2 [25].

^b^Mean difference between pre-SET intake and post-SET intake.

^c^Difference in number achieving AMDR or SDT pre-SET and post-SET.

### Alcohol

The mean (SD) alcohol intake for participants with IC was 8.6 g (13.1) pre-SET and 8.1 g (13.5) post-SET, P = 0.459. 26/31 (84%) of participants with IC achieved the recommended <2 standard drinks daily pre-SET and for those with post-SET data available, 27/29 (93%) achieved the recommendation, P = 0.025. Of those without PAD, mean (SD) alcohol intake was 8.2 g (10.0) and 18/23 (78%) achieved the recommendation for alcohol intake and this was not statistically significant compared to participants with IC pre-SET (P = 0.545).

## Discussion

The findings of this study demonstrate that dietary habits of middle-aged to older adults are poor irrespective of whether or not they are affected by PAD. A lifestyle intervention in the form of a supervised exercise program may be able to facilitate improvement in dietary intake with implementation of formal and targeted nutrition education of a greater magnitude than that provided by the current service.

Consistent with findings from Gardner et al. [8], the majority of participants with IC in the present study failed to achieve the recommended dietary targets across all key macro and micro nutrients with the exception of protein. This translates to excess total fat, cholesterol and sodium intake and sub-optimal fibre and antioxidant intake, the culmination of which is likely to favour the progression of CVD risk factors, endothelial dysfunction and subsequently the systemic burden of atherosclerosis. Added to this is the inappropriately low consumption of other nutrients such as folate which may precipitate an anaemic state and worsen the symptoms associated with IC.

Unique to this paper, an assessment of alcohol intake was also conducted demonstrating the presence of excessive levels of consumption among some participants with IC pre-SET and a spontaneous reduction culminating in more participants achieving the recommendations post-SET. While not an established risk factor for PAD, the adverse health outcomes of excess alcohol consumption are well documented and according to the World Health Organisation, alcohol is the third most harmful risk factor for chronic disease onset and progression [29].

Surprisingly, the dietary intake data from non-PAD controls demonstrated equally poor dietary intake compared to participants with IC. Given that poor diet is known to impact on CVD risk factors and disease progression, one would expect patients with IC to have a worse diet than non-PAD controls. A possible explanation for the finding in our study is that participants with IC may have had worse dietary intakes before diagnosis and subsequent referral to a specialist service and have therefore already made significant spontaneous changes to bring them in line with non-PAD controls. The timing between diagnosis and dietary assessment however lies outside the scope of this study so we cannot be definitive in this regard. Alternatively, our findings may reinforce the recently established link between genetics and progression to PAD [30]. That is, the genetic make-up of an individual is ultimately responsible for coordinating the extent of interplay between independent risk factors such as dietary intake and hypertension in order to determine extent to which such risk factors are converted to established arterial disease.

Significantly, this study is the first of its kind to assess change in dietary intake in adults with IC following SET and minimal nutrition education. Small improvements were identified in the number of participants achieving the recommended intake of sodium, cholesterol and vitamin C, however, such improvements were not observed among other nutrients. This seems to be a common trend with dietary change beyond fat, cholesterol and sodium being uncommon, as is evidenced by studies assessing the impact of exercise intervention on dietary intake in other patient groups [13–15]. While the clinical implications of such small changes are unclear, the demonstrated willingness to change dietary intake displayed by these participants as a result of an exercise intervention and minimal nutrition education provides a framework on which, given an injection of sufficient resources, an appropriately credentialed clinician could capitalise and potentially achieve clinically meaningful improvements in outcomes. Better nutrition education may facilitate improvements in the intake of a greater array of relevant nutrients including omega-3 fatty acids, anti-oxidants and fibre. This could therefore be considered as an opportunity to seize the teachable moment with the potential to improve both long and short term health outcomes in this cohort of patients who are already at a high risk of future CVD morbidity and mortality. A targeted intervention in the future to assess such an area may therefore be warranted.

In addition to exploring an array of macro and micro nutrient intake in the present study we also explored total energy intake of participants with IC. While it might be expected that an exercise program would increase total energy requirements and participants would increase energy intake to compensate for these increased demands, this was not demonstrated in the present study. Rather, energy intake remained consistent for participants with IC from pre-SET to post-SET. Interestingly this trend has in fact been reported previously by Speakman and Selman [31] who demonstrated that while exercise training can lead to increases in resting energy expenditure, compensation occurs via reducing body weight and other components of energy expenditure rather than increasing dietary energy intake. It must be highlighted that the present study did identify a high level of underreporting of energy intake and thus this area warrants further investigation. The implications of the underreporting of energy intake in the present study are important to consider in the context of the findings of the present study. Given the present study demonstrated that few participants achieved the AMDR for total fat and saturated fat or the SDTs for cholesterol and sodium, then a higher energy intake than currently reported would likely result in fewer participants having achieved the dietary targets for these critical nutrients. Conversely, if participants truly had a higher energy intake than reported then intake of nutrients including fibre, vitamin E, folate and vitamin C would have been underestimated.

Some additional limitations that warrant consideration include the sample size, method of assessing dietary intake and the lack of capturing data on complementary and alternative medicines containing key nutrients. While the sample size is not dissimilar to the only other similar study in this area [9], ideally a larger study would be conducted to confirm the findings presented in this study. The method of dietary assessment was via a food frequency questionnaire and, although comprehensively validated, food frequency questionnaires have been demonstrated to underestimate dietary intake in some settings. The food frequency questionnaire also does not allow for capturing nutritional supplements, nor did we specifically ask about these and hence, once again, the estimates of some nutrients may be underestimated in the present study. Further research in this area should attend to this deficit in methodology for the purpose of attaining a more accurate determination of nutrient intake that includes both food and supplements.

## Conclusions

In conclusion, the findings of the present study raise several questions which can be considered further in future studies. The present study has established that this high risk group with IC has sub-optimal dietary intake of an array of relevant nutrients. Although small, some spontaneous change occurs with SET and minimal nutrition education providing an indication that there may be a willingness to modify dietary behaviours. An opportunity therefore exists to consolidate nutrition services in this area in order to avoid a missed opportunity to achieve significant short and long term health benefits in a nutritionally vulnerable patient group with potentially much to gain.

## References

[1] Criqui M, Langer R, Fronek A, Feigelson HS, Klauber MR, McCann TJ, Browner D (1992). Mortality over a period of 10 years in patients with peripheral arterial disease. N Eng J Med.

[2] Norgren L, Hiatt WR, Dormandy JA, Nehler MR, Harris KA, Fowkes FG, TASC II Working Group (2007). Inter-Society Consensus for the Management of Peripheral Arterial Disease (TASC II). J Vasc Surg.

[3] Murabito JM, Evans JC, Nieto K, Larson MG, Levy D, Wilson PW (2002). Prevalence and clinical correlates of peripheral arterial disease in the Framingham Offspring Study. Am Heart J.

[4] Gattone M, Giannuzzi P (2006). Interventional strategies in early atherosclerosis. Monaldi Arch Chest Dis.

[5] Lane JS, Magno CP, Lane KT, Chan T, Hoyt DB, Greenfield S (2008). Nutrition impacts the prevalence of peripheral arterial disease in the United States. J Vasc Surg.

[6] Delaney C, Spark J, Thomas J, Wong YT, Chan LT, Miller MD (2013). A systematic review to evaluate the effectiveness of carnitine supplementation in improving walking performance among individuals with intermittent claudication. Atherosclerosis.

[7] Carrero JJ, Lopez-Huertas E, Salmeron LM, Baró L, Ros E (2005). Daily supplementation with (n-3) PUFAs, oleic acid, folic acid, and vitamins B-6 and E increases pain-free walking distance and improves risk factors in men with peripheral vascular disease. J Nutr.

[8] Gardner AW, Bright BC, Ort KA, Montgomery PS (2011). Dietary intake of participants with peripheral artery disease and claudication. Angiology.

[9] Shephard RJ, Balady GJ (1999). Exercise as cardiovascular therapy. Circulation.

[10] Leng GC, Fowler B, Ernst E (2000). Exercise for intermittent claudication. Cochrane Database Syst Rev.

[11] Watson L, Ellis B, Leng GC (2008). Exercise for intermittent claudication. Cochrane Database Syst Rev.

[12] Hiatt W, Regensteiner J, Hargarten M, Wolfel EE, Brass EP (1990). Benefit of exercise conditioning for patients with peripheral arterial disease. Circulation.

[13] Miller WC, Lindeman AK, Wallace J, Niederpruem M (1990). Diet composition, energy intake, and exercise in relation to body fat in men and women. Am J Clin Nutr.

[14] Tremblay A, Almeras N (1995). Exercise, macronutrient preferences and food intake. Int J Obes Relat Metab Disord.

[15] Wood PD, Terry RB, Haskell WL (1985). Metabolism of substrates: diet, lipoprotein metabolism, and exercise. Fed Proc.

[16] Delaney CL, Miller MD, Chataway TK, Spark JI (2014). A randomised controlled trial of supervised exercise regimens and their impact on walking performance, skeletal muscle mass and calpain activity in patients with intermittent claudication. Eur J Vasc Endvasc Surg.

[17] Montgomery P, Gardner A (1998). The clinical utility of a six-minute walk test in peripheral arterial occlusive disease patients. J Am Geriatr Soc.

[18] Giles G, Ireland P: *(Internet). User Information Guide: Dietary Questionnaire for Epidemiological Studies Version 2*. Melbourne, Victoria: The Cancer Council Victoria; Available from: http://www.cancervic.org.au/research/epidemiology/nutritional_assessment_services

[19] Xinying PX, Noakes M, Keogh J (2004). Can a food frequency questionnaire be used to capture dietary intake data in a 4 week clinical intervention trial?. Asia Pac J Clin Nutr.

[20] Lewis J, Hunt A, Milligan G (1995). NUTTAB95 Nutrient Data Table for Use in Australia.

[21] Holland B, Welch A, Unwin I, Buss DH, Paul AA, Southgate DAT (1993). McCance and Widdowson's the Composition of Foods.

[22] USDA-NCC (1998). USDA-NCC Carotenoid Database for U.S. Foods.

[23] Foster-Powell K, Holt SH, Brand-Miller JC (2002). International table of glycemic index and glycemic load values: 2002. Am J Clin Nutr.

[24] RMIT: *RMIT Fatty Acid Database of Australian Foods*. Brisbane: Xyris Software; Available from http://www.xyris.com.au

[25] National Health and Medical Research Council (2006). Nutrient Reference Values for Australia and New Zealand, Including Recommended Dietary Intakes.

[26] Schofield W (1985). Predicting basal metabolic rate, new standards and review of previous work. Hum Nutr: Clin Nutr.

[27] Black A, Cole T (2001). Biased over- or under- reporting is characteristic of individuals whether over time or by different assessment methods. J Am Dietet Ass.

[28] National Health and Medical Research Council (2009). Australian Guidelines to reduce health risks from drinking alcohol.

[29] World Health Organization (2011). Global status report on alcohol and health.

[30] Prushik S, Farber A, Gona P, Shrader P, Pencina MJ, D'Agostino RB, Murabito JM (2012). Parental Intermittent Claudication as Risk Factor for Claudication in Adults. Am J Cardiol.

[31] Speakman J, Selman C (2003). Physical activity and resting metabolic rate. Proc Nutr Soc.

